# Impact of emergency department-to-intensive care unit transfer time on in-hospital mortality: A retrospective cohort study

**DOI:** 10.1097/MD.0000000000046724

**Published:** 2025-12-19

**Authors:** Sefer Özkaya, Mehmet Serkan Yurdakul

**Affiliations:** a Karaman Training and Research Hospital, Karaman, Turkey.

**Keywords:** critical care access, ED boarding time, emergency department, ICU transfer delay, in-hospital mortality

## Abstract

We evaluated whether emergency department (ED)-to-intensive care unit (ICU) transfer time is associated with in-hospital mortality among adults admitted with ICU-level indications, using a prespecified 4-hour quality threshold. In this retrospective cohort from a tertiary academic hospital (January–December 2023), we analyzed 376 adults admitted to the ICU from the ED. Demographics, comorbidities, primary diagnoses, laboratory markers, and ED process metrics were extracted. ED length of stay (ED-LOS) was examined continuously and categorically (≤4 vs >4 hours). Multivariable logistic regression identified independent predictors of mortality. Overall in-hospital mortality was 54.3%. Median ED-LOS was 194 minutes in survivors versus 220 minutes in non-survivors. While continuous ED-LOS was not significantly different between groups, ED-LOS > 4 hours was independently associated with increased mortality (odds ratio, 1.78; 95% confidence interval, 1.13–2.82; *P* = .01). Additional predictors included advanced age, sepsis diagnosis, low serum albumin, and acute physiology and chronic health evaluation II ≥ 35; presentation during daytime hours was associated with reduced mortality (odds ratio, 0.54; 95% confidence interval, 0.30–0.97; *P* = .04). ED-to-ICU transfer delays > 4 hours are independently associated with higher in-hospital mortality. While timely ICU admission is critical, optimizing care during ED boarding may mitigate risk. These findings support system-level interventions targeting ICU transfer within 4 hours for critically ill patients.

## 1. Introduction

Emergency departments (EDs) increasingly serve as critical care entry points, particularly in settings with limited intensive care unit (ICU) capacity. In this context, ED boarding—defined as the time critically ill patients remain in the ED after the decision to admit to ICU—has emerged as a key performance indicator and patient-safety concern.^[1,2]^ The delay in transferring patients from the ED to the ICU, known as ED length of stay (ED-LOS) or more specifically ED-to-ICU transfer time, may contribute to suboptimal clinical outcomes by prolonging the time to definitive critical care management.^[3]^

Several multicenter and single-center studies have linked prolonged ED-LOS to increased mortality, length of ICU stay, and worse clinical trajectories, particularly in high-risk groups such as patients with sepsis, respiratory failure, or circulatory shock.^[4,5]^ A stepwise increase in-hospital mortality among patients with ICU transfer delays exceeding 6, 12, and 24 hours was reported.^[6]^ Patients with ED-LOS > 4 hours have significantly higher ICU and hospital mortality compared to those transferred earlier.^[7]^

In contrast, other studies have not observed a significant relationship between ED-to-ICU transfer time and survival, especially when early resuscitative care is effectively delivered in the ED; there were reported no difference in ICU mortality between early and late, accordingly, only the most severely ill patients exhibited worse outcomes with longer transfer times, and even then, the relationship was attenuated after adjusting for triage priority and acute physiology and chronic health evaluation II (APACHE II) score.^[5,8,9]^ These conflicting findings suggest that the impact of ED-LOS on patient outcomes may depend on institutional factors, including ICU triage protocols, ED staffing, and the presence of early intervention teams. While timely ICU admission is ideal, the delivery of high-quality critical care during the ED boarding phase may mitigate risks associated with transfer delays.^[10]^ Nonetheless, prolonged ED boarding is widely regarded as a system inefficiency that may expose critically ill patients to fragmented care, care delays, and ED resource strain.^[2,4]^

The present study aimed to evaluate the association between ED-to-ICU transfer time and in-hospital mortality among patients with confirmed ICU-level indications.

## 2. Methods

### 2.1. Study design and setting

This retrospective observational study was conducted at the ED of a tertiary care academic hospital between January 1 and December 31, 2023. The hospital is a regional referral center with a high-volume ED that provides 24/7 emergency and critical care services. The study protocol was reviewed and approved by the Karamanoğlu Mehmetbey University Faculty of Medicine Ethics Committee (decision no. 18-2025/01, meeting date April 9, 2025). Informed consent was not required owing to the retrospective design and use of de-identified, routinely collected data.

### 2.2. Study population

**Figure 1.**
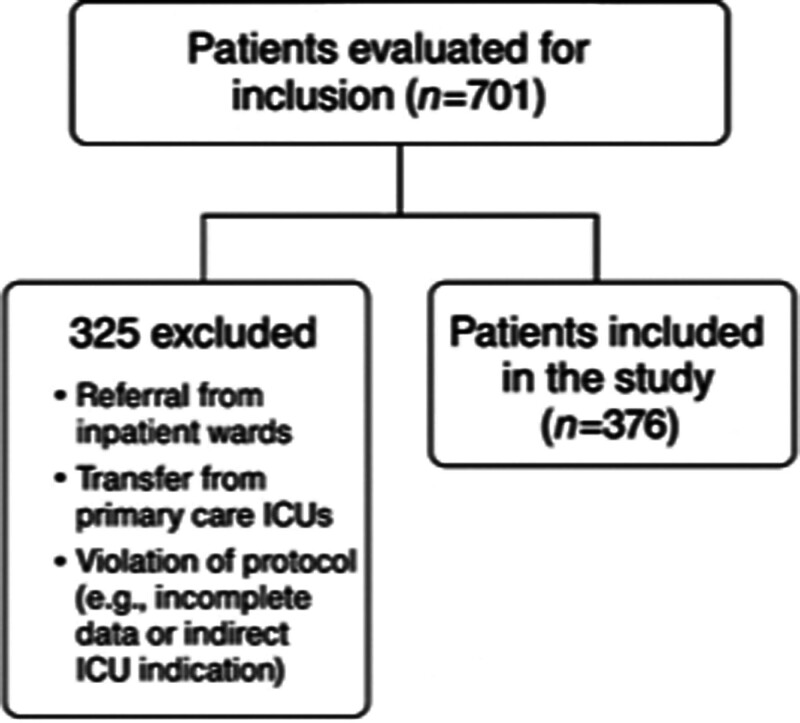
Study flow for a single-center retrospective cohort of adult emergency department (ED) patients admitted to the intensive care unit (ICU) between January and December 2023. From all ED presentations assessed for ICU-level care (n = 701), we excluded transfers from wards/other hospitals, patients without ICU-level indications, and records with missing key data (n = 325), yielding the final cohort (n = 376). Exposure: ED-to-ICU transfer time (ED length of stay, ED-LOS), analyzed continuously and dichotomized at 4 hours (≤4 vs >4). Primary outcome: in-hospital mortality. ED = emergency department, ICU = intensive care unit, IQR = interquartile range, LOS = length of stay.

The study cohort included adult patients (aged ≥ 18 years) who presented to the ED during the study period and were assessed to have an ICU admission indication. Patients were included if they were admitted to the ICU following ED evaluation and had complete clinical, laboratory, and outcome data. Exclusion criteria were: ICU transfers from wards or other hospitals; patients not meeting ICU-level clinical criteria; and missing key data elements. The process of patient screening, exclusion criteria, and final inclusion is illustrated in Figure 1.

### 2.3. Data collection and variables

Demographic characteristics (age, sex), comorbid conditions (hypertension, diabetes mellitus, chronic kidney disease, heart failure, malignancy, and cerebrovascular disease), and primary ED diagnosis were extracted from electronic medical records.

Emergency department process metrics included: ED-LOS: Defined as the time (in minutes and hours) from ED arrival to ICU transfer. ED shift: Time of ED presentation categorized as night (00:00–08:00), day (08:00–16:00), or evening (16:00–00:00). ED-LOS category: Dichotomized as ≤4 hours versus >4 hours (based on the 240-minute threshold).

Clinical severity was assessed using APACHE II score, recorded at ICU admission. ICU LOS: Recorded in days. Mechanical ventilation requirement: Use of invasive mechanical ventilation during ICU stay. Vasopressor support: Administration of any vasopressor for circulatory support.

The primary outcome was in-hospital mortality, defined as death during the same hospital admission following ED presentation and ICU transfer.

### 2.4. Statistical analysis

All analyses were performed using SPSS version 27. Categorical variables were summarized as frequencies and percentages and compared between groups using the chi-square or Fisher exact test, as appropriate. Continuous variables were expressed as mean ± standard deviation or median with interquartile range (IQR), depending on normality (assessed using the Shapiro–Wilk test). Between-group comparisons of continuous variables were made using Student *t* test or Mann–Whitney *U* test. ED-LOS and presentation shift were analyzed both as continuous and categorical variables. Effect sizes were reported as: Cohen *d* for continuous variables (small: 0.2; medium: 0.5; large: 0.8), Cramér *V* or *φ* (phi coefficient) for categorical variables (small: 0.1; medium: 0.3; large: 0.5). A 2-tailed *P*-value < .05 was considered statistically significant.

## 3. Results

A total of 376 clinical episodes were analyzed. The median age of the cohort was 74.00 years (IQR 64.00–84.00), and 52.7% of the patients were male. Hypertension (64.4%), diabetes (37.8%), and heart failure (34.6%) were the most prevalent comorbidities. Malignancy and chronic kidney disease were more common among survivors, whereas sepsis and cardiovascular events were more frequent among non-survivors. The most common primary diagnosis was severe pneumonia (47.9%), followed by sepsis (16.5%) and cardiovascular conditions excluding myocardial infarction (13.3%). The median ICU length of stay was 7.00 days (IQR 2.00–20.5), with longer durations among survivors (Table 1).

**Table 1:** Baseline demographic, comorbidity, and diagnostic characteristics of the ICU-indicated episodes.

Characteristic	Total (n = 376)	Survivors (n = 172)	Non-survivors (n = 204)	*P*-value
Age (yr), median [IQR]	74.00 [64.00–84.00]	76.50 [67.00–85.00]	73.00 [59.25–81.75]	<.001*
Gender (male: female)	198 (52.66%) male	94 (54.65%) male	104 (50.98%) male	.54
	178 (47.34%) female	78 (45.35%) female	100 (49.02%) female	
Comorbid conditions				
Hypertension	242 (64.36%)	120 (69.77%)	122 (59.80%)	.06
Diabetes mellitus	142 (37.77%)	70 (40.70%)	72 (35.29%)	.33
CKD	54 (14.36%)	32 (18.60%)	22 (10.78%)	.05
Heart failure	130 (34.57%)	62 (36.05%)	68 (33.33%)	.66
Malignancy (active cancer)	54 (14.36%)	38 (22.09%)	16 (7.84%)	<.001
History of cerebrovascular event	98 (26.06%)	46 (26.74%)	52 (25.49%)	.87
Primary diagnosis				
Severe pneumonia (with respiratory failure)	180 (47.87%)	76 (44.19%)	104 (50.98%)	.17
Sepsis	62 (16.49%)	20 (11.63%)	42 (20.59%)	.02
Cardiovascular event (non-MI)	50 (13.30%)	16 (9.30%)	34 (16.67%)	.05
Acute myocardial infarction (MI)	2 (0.53%)	0 (0.00%)	2 (0.98%)	.50
Intoxication	36 (9.57%)	30 (17.44%)	6 (2.94%)	<.001
Neurologic event	36 (9.57%)	12 (6.98%)	24 (11.76%)	.10
Organ failure	10 (2.66%)	8 (4.65%)	2 (0.98%)	.05
ICU length of stay (days), median [IQR]	7.00 [2.00–20.50]	12.00 [3.00–28.00]	6.00 [2.00–11.00]	<.001

Values are given as median [IQR] for continuous variables and number (percentage) for categorical variables. Counts and percentages for survivor and non-survivor columns correspond to episodes grouped by final survival status.

AKI = acute kidney injury, CKD = chronic kidney disease, DKA = diabetic ketoacidosis, ICU = intensive care unit, IQR = interquartile range, MI = myocardial infarction, MOF = multi-organ failure. Comorbid conditions refer to preexisting diagnoses. Neurologic events include acute cerebrovascular accidents, seizures (status epilepticus), and central nervous system infections (e.g. meningitis).

* *P*-value for age comparison by Mann–Whitney *U* test.

Admission laboratory values did not significantly differ between survivors and non-survivors in terms of white blood cell count, platelet count, creatinine, or lactate. However, non-survivors had lower serum albumin levels (29.4 ± 6.4 g/L) compared to survivors (34.7 ± 6.1 g/L, *P* < .001), while C-reactive protein (CRP) and CRP/albumin ratio were significantly higher in survivors. Total bilirubin levels were higher in survivors (median 0.75 mg/dL) compared to non-survivors (0.54 mg/dL, *P* = .01). Mechanical ventilation and vasopressor use were recorded more frequently among survivors (Table 2).

**Table 2:** Admission laboratory parameters and clinical scoring on admission.

Parameter	Survivors (n = 172)	Non-survivors (n = 204)	*P*-value
WBC (×10^9^/L), median [IQR]	12.70 [8.10–16.90]	13.00 [9.30–16.80]	.48
Platelets (×10^9^/L), median [IQR]	232 [160–316]	242 [187–301]	.44
Creatinine (mg/dL), median [IQR]	1.35 [0.86–2.24]	1.17 [0.92–1.57]	.32
Total bilirubin (mg/dL), median [IQR]	0.75 [0.49–1.10]	0.54 [0.38–0.90]	.01
Albumin (g/L), mean ± SD	34.70 ± 6.10	29.40 ± 6.40	<.001
Lactate (mmol/L), median [IQR]	2.40 [1.51–4.94]	2.30 [1.73–4.29]	.81
CRP (mg/L), median [IQR]	89.00 [24.00–145.20]	48.20 [7.40–120.60]	.02
CRP/Albumin ratio, median [IQR]	2.80 [0.82–5.72]	1.50 [0.21–4.00]	<.001
APACHE II category (score range)			<.001
<15 (low)	6 (15.0%)	34 (85.0%)	
15–24 (moderate)	12 (25.0%)	36 (75.0%)	
25–34 (high)	20 (43.5%)	26 (56.5%)	
≥35 (very high)	134 (55.4%)	108 (44.6%)	
Mechanical ventilation required—n (%)	44 (25.58%)	28 (13.73%)	<.001
Vasopressor use—n (%)	22 (12.79%)	12 (5.88%)	.03

Continuous variables are given as median [IQR] or mean ± SD as appropriate; categorical data are given as n (%).

*P*-values reflect comparisons between survivor and non-survivor groups.

APACHE II = acute physiology and chronic health evaluation II, CRP = C-reactive protein, IQR = interquartile range, SD = standard deviation, WBC = white blood cell count.

The mean ED-LOS for the entire cohort was 236.9 ± 179.1 minutes, with no statistically significant difference between survivors and non-survivors in terms of continuous ED-LOS (both in minutes and hours). However, a categorical comparison revealed a higher proportion of survivors with ED-LOS ≤ 4 hours (72.1%) compared to non-survivors (58.8%, *P* = .01). Shift of ED presentation also varied significantly: survivors more commonly presented during the day, whereas non-survivors were more likely to present overnight (Table 3).

**Table 3:** Emergency department length of stay, shift timing, and boarding category of the patients.

Variable	Overall (n = 376)	Survivors (n = 172)	Non-survivors (n = 204)	*P*-value	Effect size
ED-LOS (min)	236.90 ± 179.10; 194 (129–271)	211.20 ± 124.90; 186 (135–256)	258.60 ± 212.30; 220 (120–311)	.30	*d* = 0.27*
ED-LOS (h)	3.9 ± 3.0; 3.0 (2–5)	3.5 ± 2.1; 3.0 (2–4)	4.3 ± 3.5; 4.0 (2–5)	.30	*d* = 0.27*
Presentation shift	1: 50 (13.30%); 2: 158 (42.02%); 3: 168 (44.68%)	1: 16 (9.30%); 2: 86 (50.00%); 3: 70 (40.70%)	1: 34 (16.67%); 2: 72 (35.29%); 3: 98 (48.04%)	<.001	*V* = 0.16†
ED-LOS category	1: 244 (64.89%); 2: 132 (35.11%)	1: 124 (72.09%); 2: 48 (27.91%)	1: 120 (58.82%); 2: 84 (41.18%)	.10	*φ* = 0.13†

ED-LOS values are shown in both minutes and hours (mean ± SD; median [IQR]). Presentation shift categories: 1 = night, 2 = day, and 3 = evening. ED-LOS category: 1 = short stay (≤4 h), 2 = prolonged stay (>4 h). Effect sizes are given as Cohen *d* for continuous variables and Cramér *V (φ* for 2 × 2) for categorical comparisons.

ED = emergency department, ED-LOS = emergency department length of stay, IQR = interquartile range, SD = standard deviation.

* Cohen *d* (effect size for difference in means): ~0.2 = small, ~0.5 = medium, and ~0.8 = large.

† Cramér *V* (for ≥ 3 categories) and *φ* (phi, for 2 categories) are effect size measures for categorical associations: ~0.1 = small, ~0.3 = medium, and ~0.5 = large. Presentation shift 1 = 00:00–08:00 (night), 2 = 08:00–16:00 (day), and 3 = 16:00–00:00 (evening). ED-LOS category 1 = ≤240 min (4 h), 2 = >240 min.

Multivariate logistic regression analyses were performed to identify independent predictors of in-hospital mortality across 3 distinct models incorporating demographic/diagnostic variables, laboratory/severity parameters, and ED process metrics.

In the model including demographic and diagnostic variables, advanced age was independently associated with increased mortality (odds ratio [OR], 1.02, 95% confidence interval [CI], 1.00–1.03, *P* = .04). Presence of active malignancy (OR, 0.29, 95% CI, 0.13–0.63, *P* < .001) and intoxication as the primary diagnosis (OR, 0.15, 95% CI, 0.04–0.55, *P* < .001) were associated with decreased odds of death. Sepsis diagnosis was positively associated with mortality (OR, 2.22, 95% CI, 1.15–4.27, *P* = .02).

In the laboratory and severity score model, higher serum albumin level (OR, 0.90, 95% CI, 0.84–0.96, *P* < .001), higher total bilirubin (OR, 0.60, 95% CI, 0.36–0.98, *P* = .04), and higher CRP/albumin ratio (OR, 0.92, 95% CI, 0.85–0.99, *P* = .04) were independently associated with reduced risk of in-hospital mortality. In multivariate analysis, higher total bilirubin remained independently associated with reduced risk of in-hospital mortality (OR, 0.60, 95% CI, 0.36–0.98, *P* = .04). An APACHE II score ≥ 35 was associated with higher mortality (OR, 3.38, 95% CI, 1.29–8.84, *P* = .01). The need for mechanical ventilation (OR, 2.44, 95% CI, 1.26–4.73, *P* < .001) and vasopressor support (OR, 2.25, 95% CI, 1.08–4.68, *P* = .03) were also independently associated with increased odds of death.

In the model based on ED process variables, ED-LOS > 4 hours was independently associated with increased mortality (OR, 1.78, 95% CI, 1.13–2.82, *P* = .01). Daytime presentation (08:00–16:00) was associated with lower mortality compared to night shift (00:00–08:00) (OR, 0.54, 95% CI, 0.30–0.97, *P* = .04). No significant association was found for evening shift presentation (OR, 0.77, 95% CI, 0.45–1.30, *P* = .31).

All model statistics including ORs, 95% CIs are presented in Table 4.

**Table 4:** Independent predictors of in-hospital mortality after ICU transfer.

Predictors*	β coefficient	SE	OR (Exp(β))	95% CI for OR	*P*-value
Age (per yr)	0.015	0.008	1.02	1.00–1.03	**.04**
Male gender	‐0.21	0.25	0.81	0.49–1.34	.40
Hypertension	‐0.12	0.28	0.88	0.51–1.52	.65
Diabetes mellitus	‐0.18	0.27	0.84	0.49–1.44	.53
CKD	‐0.52	0.35	0.60	0.30–1.20	.15
Heart failure	0.10	0.29	1.11	0.63–1.96	.72
Malignancy	‐1.25	0.39	0.29	0.13–0.63	**<.001**
Cerebrovascular disease	‐0.03	0.29	0.97	0.55–1.69	.92
Sepsis	0.80	0.34	2.22	1.15–4.27	**.02**
Severe pneumonia	0.24	0.29	1.27	0.72–2.24	.41
Cardiovascular event (non-MI)	0.62	0.33	1.86	0.97–3.54	.06
Acute MI	1.62	1.21	5.05	0.49–52.0	.18
Intoxication	‐1.90	0.65	0.15	0.04–0.55	**<.001**
Neurologic event	0.34	0.36	1.41	0.69–2.89	.34
Organ failure	‐1.20	0.81	0.30	0.06–1.50	.14
Predictors**	β coefficient	SE	OR (Exp(β))	95% CI	*P*-value
WBC	0.01	0.03	1.01	0.96–1.06	.68
Platelet	‐0.001	0.001	0.999	0.997–1.001	.23
Creatinine	‐0.14	0.16	0.87	0.63–1.19	.39
Total bilirubin	‐0.52	0.25	0.60	0.36–0.98	**.04**
Albumin (g/L)	‐0.10	0.03	0.90	0.84–0.96	**<.001**
Lactate	0.05	0.06	1.05	0.93–1.17	.41
CRP/Alb ratio	‐0.08	0.04	0.92	0.85–0.99	**.04**
APACHE moderate (15–24)	0.31	0.53	1.36	0.48–3.89	.57
APACHE high (25–34)	0.64	0.51	1.89	0.70–5.10	.21
APACHE very high (≥35)	1.22	0.49	3.38	1.29–8.84	**.01**
Mechanical ventilation	0.89	0.34	2.44	1.26–4.73	**<.001**
Vasopressor use	0.81	0.38	2.25	1.08–4.68	**.03**
Predictors***	β coefficient	SE	OR (Exp(β))	95% CI	*P*-value
ED-LOS > 4 h	0.58	0.23	1.78	1.13–2.82	**.01**
Shift—day	‐0.61	0.28	0.54	0.30–0.97	**.04**
Shift—evening	‐0.26	0.26	0.77	0.45–1.30	.31

Bold values indicate statistical significance (*P* < .05).

APACHE II = acute physiology and chronic health evaluation II, AUC = area under the curve, CKD = chronic kidney disease, CRP = C-reactive protein, ED = emergency department, ED-LOS = emergency department length of stay, MI = myocardial infarction, OR = odds ratio, ROC = receiver operating characteristic, SE = standard error, WBC = white blood cell count.

* *The demographic and diagnostic model*: Hosmer–Lemeshow test: *P* = .64, indicating good model calibration; Nagelkerke *R*^2^ = 0.21; area under the ROC curve (AUC) = 0.75, reflecting acceptable discriminative capacity.

** *The laboratory and severity score model*: Hosmer–Lemeshow test: *P* = .53, confirming good model fit; Nagelkerke *R*^2^ = 0.29; AUC = 0.78, indicating good discrimination.

*** *The emergency department process model* (): Hosmer–Lemeshow test: *P* = .70, Nagelkerke *R*^2^ = 0.09 (reflecting limited explanatory power due to process-only variables); AUC = 0.67, consistent with modest discriminative ability, though independent effects remained evident.

## 4. Discussion

In this single-center cohort study, prolonged ED-LOS prior to ICU transfer was significantly associated with higher in-hospital mortality. Patients who remained in the ED for more than 4 hours before ICU admission had nearly twice the odds of dying in the hospital compared to those transferred within 4 hours (OR, 1.78). This association persisted even when accounting for other factors, underscoring ED boarding time as an independent risk factor. In addition to transfer delays, we observed that patients presenting during daytime hours experienced lower mortality than those arriving overnight (OR, 0.54; 95% CI, 0.30–0.97), suggesting that resource availability or staffing levels could influence outcomes. No significant difference was seen for evening versus night presentations. Taken together, these findings highlight timely ICU admission as a critical determinant of survival and point to possible diurnal variations in care that warrant further attention.

Our results align with a growing body of literature indicating that delays in transferring critically ill patients from the ED to the ICU can adversely affect outcomes. Multiple prior studies have documented that longer ED boarding times correlate with increased mortality, longer ICU stays, and more frequent complications in high-risk populations such as those with sepsis, respiratory failure, or shock.^[2–4,11,12]^ Some reports even describe a stepwise rise in mortality risk as ED-LOS extends into extreme ranges, reinforcing the concept that the longer a critically ill patient waits for ICU-level care, the greater the potential harm.^[4,8]^ Conversely, not all investigations have found a uniform relationship between ED transfer time and survival. Notably, a large multicenter study in the Netherlands observed that only the most severely ill patients, as indicated by high APACHE scores, showed worsened outcomes with longer ED stays, and even then the effect was attenuated after adjusting for illness severity and triage priority.^[12–14]^ Other single-center analyses have reported no significant mortality difference between early and delayed ICU admissions when robust resuscitative treatment was delivered in the ED during the wait.^[1]^ These disparate findings suggest that context matters: the impact of ED boarding on mortality may depend on institutional and operational factors. Differences in ICU triage protocols, availability of ICU beds, ED staffing levels, especially the presence of experienced critical care personnel during off-hours, and the use of dedicated ED-based critical care teams can all modulate how a transfer delay translates into patient outcome. Overall, however, the preponderance of evidence—including recent meta-analyses—indicates that prolonged ED lengths of stay are detrimental, particularly once delays become excessive. Our study adds to this evidence by demonstrating a significant mortality increase at a relatively early threshold of 4 hours, emphasizing that even moderate delays should not be dismissed as inconsequential.

The identification of a 4-hour ED-LOS threshold associated with increased mortality carries important clinical and operational implications. Four hours is a timeframe often cited in emergency care benchmarks.^[15,16]^ Our findings specifically validate this cutoff in the context of critically ill patients requiring ICU admission. In practical terms, a 4-hour window may represent a critical period within which ICU transfer should ideally occur to optimize survival chances. Beyond this point, patients may be more likely to deteriorate or experience delays in definitive therapies, such as advanced hemodynamic monitoring, organ support, or specialist interventions available only in the ICU setting.^[17,18]^ Hospitals should therefore consider 4 hours as an achievable and meaningful target for ICU admission decisions and logistics, focusing improvement efforts on expediting transfers within this interval whenever possible. It is also worth noting that the capacity of the ED to function as an extended critical care unit can influence outcomes.^[19,20]^ Our data and prior studies suggest that when EDs are equipped to provide high-quality critical care—including timely sepsis management, mechanical ventilation, vasopressors, and close monitoring—they can partly buffer the negative effects of transfer delays.^[3,12]^ In other words, effective early resuscitation and stabilization in the ED may “buy time” for patients who cannot be transferred immediately. However, this should not be viewed as a permanent solution or justification for routine boarding of ICU-bound patients. Even with excellent ED care, the emergency department is not designed for prolonged ICU-level management: space, staffing, and resource constraints make extended boarding a suboptimal substitute for an ICU bed. Prolonged ED stays for critically ill patients are therefore best regarded as a system inefficiency—one that exposes patients to potential lapses in optimal care continuity, prolongs pain and uncertainty, and creates bottlenecks that can strain ED capacity for new emergencies. Our findings reinforce the need to address ED-to-ICU transfer delays not only as a matter of individual patient risk but also as a target for system-wide quality improvement.

### 4.1. Study limitations

Limitations of this study should be acknowledged when interpreting results. First, data are drawn from a single tertiary care center over 1 year, which may limit generalizability to other settings with different patient populations or resource levels. The retrospective design carries risk of unmeasured confounding; although we adjusted for key variables like illness severity and presentation timing, other factors could influence transfer time and outcomes. We defined a delay threshold of >4 hours based on clinical rationale and literature, but this dichotomization may oversimplify the relationship between waiting time and risk. Patients who died before ICU admission were not included, as our study population consisted only of those reaching the ICU—this could underestimate transfer delays’ impact, since extreme consequences (death in ED while awaiting ICU) were not captured. These limitations highlight the need for cautious interpretation and validation in broader contexts.

## 5. Conclusion

In this single-center retrospective cohort, longer emergency department-to-ICU transfer times were independently associated with higher in-hospital mortality after adjustment for illness severity and key confounders. Using a pragmatic 4-hour threshold, delays beyond this window identified higher-risk patients and highlighted a modifiable operational target. These findings support adopting ED-to-ICU transfer time as a quality metric, implementing escalation pathways for bed allocation, and prioritizing early ICU access for deteriorating patients. While causality cannot be inferred from an observational design, the results underscore timely transfer as a patient-safety lever aligned with process improvement. Multicenter validation across diverse settings is warranted to confirm generalizability and to set achievable benchmarks for hospital quality initiatives.

## Author contributions

**Data curation:** Sefer Özkaya.

**Investigation:** Sefer Özkaya.

**Methodology:** Sefer Özkaya.

**Project administration:** Mehmet Serkan Yurdakul.

**Software:** Mehmet Serkan Yurdakul.

**Validation:** Mehmet Serkan Yurdakul.

**Visualization:** Sefer Özkaya.

**Writing – original draft:** Sefer Özkaya.

**Writing – review & editing:** Sefer Özkaya, Mehmet Serkan Yurdakul.
